# Value set of EQ-5D-Y-3L for Hong Kong

**DOI:** 10.1186/s12955-026-02522-y

**Published:** 2026-03-23

**Authors:** Eliza Lai-Yi Wong, Kailu Wang, Amy Yuen-Kwan Wong, Annie Wai-Ling Cheung, Carlos King Ho Wong, Nan Luo, Oliver Rivero-Arias, Eng-Kiong Yeoh

**Affiliations:** 1 JC School of Public Health and Primary Care, Faculty of Medicine, The Chinese University of Hong Kong, Hong Kong SAR, China; 2 Centre for Health Systems and Policy Research, JC School of Public Health and Primary Care, Faculty of Medicine, The Chinese University of Hong Kong, Hong Kong SAR, China; 3 Department of Infectious Disease Epidemiology & Dynamics, Faculty of Epidemiology and Population Health, The London School of Hygiene and Tropical Medicine, London, UK; 4 School of Public Health, Li Ka Shing Faculty of Medicine, The University of Hong Kong, Hong Kong SAR, China; 5 Saw Swee Hock School of Public Health, National University of Singapore, Singapore, Singapore; 6 National Perinatal Epidemiology Unit, Medical Sciences Division, University of Oxford, Oxford, UK; 7 Rm 418, School of Public Health Building, Prince of Wales Hospital, Shatin, N.T Hong Kong

**Keywords:** EQ-5D-Y value set, preference weights, Discrete Choice Experiment (DCE), Composite Time Trade-Off (C-TTO), Economic evaluations

## Abstract

**Objectives:**

The EQ-5D-Y instrument is currently used to assess health-related quality of life of health states experienced by young populations. However, a country-specific EQ-5D-Y value set for Hong Kong (HK) is not yet available. The study aimed to develop an HK-specific value set for the EQ-5D-Y, following the valuation proposal recommended by the EuroQol Group.

**Methods:**

The study included a Discrete Choice Experiment (DCE) exercise to estimate the relative importance of each level within the five dimensions of the EQ-5D-Y. A composite Time-Trade Off (C-TTO) was also conducted to anchor the latent scale derived from the DCE to the quality-adjusted life years (QALY) 0–1 scale. Both tasks were administered using the EQ-PVT platform. Responses were collected from adults in the general population, who completed the elicitation tasks from a child’s perspective.

**Results:**

A total of 1,001 adult participants responded for the Discrete Choice Experiment (DCE), while 205 adults completed the Composite Time Trade-Off (C-TTO) tasks. The sample was representative of the general Chinese population in HK in terms of age, sex, and geographical distribution. Among the five EQ-5D-Y dimensions, the most influential in determining health state preferences was pain/discomfort, followed by feeling worried, sad, or unhappy; performing usual activities; mobility; and self-care.

**Conclusion:**

This study presents an EQ-5D-Y-3 L value set for the HK Chinese young population, following the international valuation protocol of this instrument. This value set enables future use of the EQ-5D-Y-3 L for measuring health-related quality of life and conducting economic evaluation for children and adolescents.

## Key points

• Among the five EQ-5D-Y dimensions, pain/discomfort emerged as the most influential factor in shaping health state preferences, followed by feeling worried, sad, or unhappy, performing usual activities, mobility, and self-care. This ranking differs from that observed in adult Chinese populations, underscoring the need for a distinct, youth-specific value set to capture health-related quality of life in children and adolescents.

• The study establishes the EQ-5D-Y-3 L value set specifically for children and adolescents in Hong Kong which can provide a robust instrument for assessing health-related quality of life among the young population.

• The instrument also enables economic evaluations of health interventions, supporting evidence-based resource allocation for the younger population within this jurisdiction.

## Introduction

In 2009, the EQ-5D-Y – a generic preference-based patient-reported instrument to measure health-related quality of life (HRQoL) was developed for children and adolescents, which was adapted from the standard adult version of EQ-5D-3L [1]. This tool is a patient-reported outcome measure (PROM) that captures the HRQoL across five dimensions: mobility, self-care, usual activities, pain/discomfort, and anxiety/depression, each assessed at three levels (no problems, some problems and extreme problems). The only difference between the adult version (EQ-5D-3L) and the youth version (EQ-5D-Y-3 L) is that the latter uses more appropriate and child-friendly wording to facilitate understanding among children and adolescents. While the EQ-5D has been utilised globally in various settings, including clinical trials and population surveys, its popularity stems from its concise design and broad applicability across diverse health conditions. The dual reporting format includes a descriptive profile of health status and an index score that reflects preference-based values, making it a versatile and practical tool for assessing HRQoL. The adapted EQ-5D-Y was initially translated into four languages: German, Italian, Spanish and Swedish, and cognitive interviews were conducted to test its acceptability and feasibility for use in youth populations. A multinational study demonstrated that EQ-5D-Y is a feasible and well-validated instrument for assessing HRQoL among children and adolescents in Western countries [2]. To facilitate its use in Chinese-speaking populations, the instrument was subsequently translated into Chinese following a standardised protocol developed by the EuroQoL Research Foundation [3]. Validation studies of the Chinese version have been conducted across various Chinese populations [4–6], confirming its reliability and applicability.

In Hong Kong (HK), PROMs are increasingly valued in promoting patient-centered-care, as they reflect the importance of the patient’s perspective in both subjective and objective ways. PROMs also measure health benefits in the economic evaluation of health technologies. Although health preference weights using the EQ-5D value set for adults were developed in HK to inform health policy [7], a similar tool for youth is lacking and applying the adult EQ-5D-3L value sets has been deemed inappropriate [8]. Health preferences are not only important for adults but also for children and adolescents, as they are healthcare service users with distinct health needs. Given the Hong Kong Special Administrative Region Government’s emphasis on youth voices in policy-making, highlighted by the establishment of the Youth Development Commission in 2018, establishing a Hong Kong-specific EQ-5D-Y value set is crucial to reflect youth preferences and guide decision-making.

Developing such a value set for younger populations posed challenges not only in translation and cultural adaptation but also in unique conceptual and methodological aspects. These include determining whose values to prioritize, the perspective to adopt, and the appropriate elicitation and anchoring methods compared to those used for adult populations [8]. With the growing global demand for value sets for the EQ-5D-Y, Kreimeier and colleagues evaluated the impact of wording and perspective (child or adult) in the valuation exercise to investigate whether these valuations of health states would be affected by methodological variations in four countries (Germany, Spain, England, and the Netherlands). The findings revealed an interaction effect between the wording of the instrument and the perspective on elicitation (child or adult). The observed discrepancies in valuation when applying the adult EQ-5D-3L and the youth EQ-5D-Y instruments indicate that adult EQ-5D-3L value sets are inappropriate for valuing EQ-5D-Y health states.

An international EQ-5D-Y valuation protocol was initiated in 2020 by The EuroQol Research Foundation, which pursued a methodological research programme to provide insights into addressing the identified conceptual and methodological challenges in eliciting EQ-5D-Y health states [9]. This protocol was rapidly adopted by local study teams worldwide. It incorporates two key valuation tasks that are identical to those used for valuing EQ-5D-5 L [10]: (1) discrete choice experiments (DCE) to determine the relative importance of the five dimensions or severity levels; and (2) the composite time-trade-off (C-TTO) to anchor the DCE values on a scale where 1 represents full health and 0 represents death [9]. The roles of DCE and C-TTO differ in EQ-5D-Y compared to valuing EQ-5D-5 L, where DCE is used as the principal method and a different perspective is adopted. For instance, adults are asked to value health states from the perspective of a 10-year-old child. While a few methodological studies have shown that the observed values differ between child and adult health states, it was concluded that adults’ self-reported health states were valued higher than those applicable to a 10-year-old child. It is acknowledged that a decision was made in the EQ-5D-Y valuation protocol to apply the taxpayer perspective when valuing child health states, as these values inform health technology assessment (HTA) and resource allocation decisions. Since DCE results are on a latent (undefined) scale, an anchoring method is required to derive value sets and C-TTO was employed for this purpose in the study. However, further research is needed to determine appropriate methods for anchoring these DCE results [11].

The EQ-5D-Y is widely used to assess HRQoL among youth populations. However, there is currently no country-specific EQ-5D-Y value set available for the local Chinese population in Hong Kong (HK). Evidence suggests that applying adult value sets for EQ-5D-Y states is inappropriate. Furthermore, HK’s distinctive status as a former British colony and current Special Administrative Region of China has resulted in a different health system, cultural norms, demographics, and socioeconomic characteristics that differ from those in mainland China [12]. These unique characteristics make it essential to develop valuation studies tailored to the local child population. This study aimed to develop a value set for the EQ-5D-Y-3 L from the perspective of the local adult population in HK, following the recommended international valuation protocol for EQ-5D-Y studies established by the EuroQol Group.

## Methods

The design of this study followed the published EQ-5D-Y valuation protocol [9], which includes a DCE for adults to obtain the relative importance of the EQ-5D-Y-3 L dimensions and levels, as well as a C-TTO to anchor the latent DCE values on a scale ranging from − 1 to 1, where 1 refers to full health and 0 refers to death.

### DCE design

The DCE for adults included 300 pairs of health states derived using a D-efficient algorithm without prior means for the five health dimensions of EQ-5D-Y. These pairs were divided into 10 blocks, each containing 15 pairs. In each pair, there were 2 dimensions at the same level and 3 dimensions with different levels between the health states, with the differing levels highlighted in bolded font. Two dominant pairs were included for all respondents (state 21212 vs. 31223 & state 31212 vs. 32332) for quality control purposes. The order of the pairs and the left-right position of the health states within each pair were randomised for different individuals. The dimensions with different levels across the two health states in the same pair were bolded in the DCE exercise to reduce the cognitive burden.

### C-TTO design

The C-TTO design involves a total of 10 health states, including 3 mild (11112, 11121, 21111), 2 moderate (22223, 22232), and 3 severe (31133, 32223, 33233, 33323, 33333) health states. Before the formal C-TTO tasks, two C-TTO examples were provided for the interviewers to illustrate and explain the tasks, and three “wheelchair” C-TTO tasks were included for respondents to practice. In addition to the C-TTO tasks, a Hong Kong Traditional Chinese version of the EQ-5D-5 L questionnaire^3^ was used to collect their quality of life. Background information on their socio-demographic characteristics and health status was also collected in the questionnaire.

### Sampling and subject recruitment

According to the standardised valuation study protocol by the EuroQol Group [9], a minimum of 1,000 adult respondents were proposed to conduct face-to-face DCE interviews using a child perspective. An additional 200 adults would be asked to complete the C-TTO interview, also using a child perspective. A representative sample was recruited using a stratified quota sampling method based on age, sex and educational attainment across three geographical areas in 18 districts of Hong Kong: Kowloon, New Territories and Hong Kong Island. The sample population was estimated using the 2021 Population By-census from the Census and Statistics Department, HKSAR [13]. Participants included Cantonese-speaking Hong Kong residents aged 18 years or older who were able to read Chinese. The study was promoted via various social media channels and an internal research hub, providing details about the research and the study procedures. Interested participants were required to register via online platform. The study team verified eligibility before study enrolment. Participants received an incentive of HKD$100 (~ USD$13) in supermarket coupons as a token of appreciation for their participation.

### Survey instrument and data collection process

Data collection through face-to-face interviews was conducted between December 2021 and July 2023. Alternative Zoom interviews were also conducted simultaneously during the COVID-19 pandemic due to policy restrictions on social distancing measures enforced by the Hong Kong SAR government. All Zoom interviews required participants to have their cameras on, which was regarded as equivalent to an in-person interview. The Zoom administration mode was confirmed to be as effective as face-to-face interviews (NRef5). The sampling quotas were monitored by the research team via an online registration system. Individuals were not invited to participate if the quota for their age, sex or educational attainment category had already been filled. All interviews were conducted and data were stored using the computer-based valuation software (EuroQoL Portable Valuation Technology, EQ-PVT) [14] developed by The EuroQol Group, in accordance with the valuation protocol, by six well-trained interviewers proficient in using the EQ-VT software. The training materials for interviewers were prepared in English by the EuroQol Group and were officially translated into Traditional Chinese for the Hong Kong Chinese version. Regarding data quality [14, 15], we recorded the time spent completing the DCE tasks and a threshold of 150 s was applied for quality control. In addition, responses were excluded if participants incorrectly answered two dominant pairs. Furthermore, respondents who provided unusual choice patterns across all DCE tasks were removed, such as consistently selecting the same state (e.g., State A) in every task. For the C-TTO exercise, we followed the standard quality control procedures developed by EuroQol, which primarily focus on interviewer behaviour and variability between interviewers [16]. All interviewers had prior experience; however, they also underwent refresher training and completed 20 pilot cases to ensure interview quality.

### Statistical analysis

Socio-demographic characteristics of the study sample were described using proportions of the categorical variables. Response behaviours, including the left-right position of the chosen alternatives and the proportion of selections for certain Eq. 5D states, were also summarised. In the C-TTO, respondents who provided inconsistent or unrealistic valuations (e.g., higher valuations for more severe states) were excluded from the analysis. Based on the C-TTO responses, the means and standard errors of the observed C-TTO values were reported separately for the 10 health states. A Tobit model with censoring at -1 was estimated to adjust the C-TTO utility, as the C-TTO does not allow utility values lower than − 1.

Respondents in the DCE who failed the dominant DCE tasks (i.e., either one of the two pairs) were excluded from the analysis. The DCE responses were first analysed using a main-effect multinomial logit model (MNL) and a mixed logit model (MIXL), with or without an alternative specific constant (ASC) to indicate whether the corresponding alternative is on the left-hand or right-hand side of the choice set. In these models, level 1 of each dimension (“no problem”) served as the reference level. The model with the lowest Bayesian Information Criterion (BIC) was selected to derive the utility index. The relative importance of each dimension was determined based on the range of the model coefficients for that dimension, where a larger range indicated greater relative importance.

Following the MIXL modelling, a linear mapping approach was used to anchor the DCE weightings into the EQ-5D scale using C-TTO responses. In this approach, the DCE weightings can be linearly rescaled by taking into account the relationship between the latent DCE utility and the observed utility of all 10 C-TTO health states. A linear regression was performed with the observed disutility values of the 10 C-TTO health states as the dependent variable and the latent DCE disutility of the 10 states as the independent variable, without a constant. The model coefficient of the latent DCE utility was considered the rescaling parameter. The MIXL model coefficients were then rescaled by this parameter to derive the EQ-5D-Y-3 L value set. Two additional anchoring approaches were also performed for comparison with the linear mapping approach: (1) worst state anchoring, which rescales based on the latent DCE utility value and the observed utility value of the worst state (33333), and (2) hybrid modelling, which estimates the utility values using a single hybrid model incorporating DCE and C-TTO responses of all 10 health states using the Stata command hyreg [17]. The model coefficients weighted by class shares were used to calculate the value set [18]. The prediction accuracy of the three anchoring approaches was measured by the mean absolute error (MAE) and the root mean square error (RMSE) between the observed utility and the value-set-based predicted utility of the 10 C-TTO health states. The anchoring approach with the lowest MAE and RSME would be chosen as the value set. The model outcomes are presented in regular dummy coding, where each coefficient represents the effect of a level relative to a single reference category (i.e., no problem). This approach was chosen to facilitate straightforward interpretation. All statistical analyses were performed using Stata SE 16.

## Results

### Sample characteristics

For the DCE, 1,030 adult participants with valid age information completed the online survey. Among them, 29 participants (2.8%) were excluded from the analysis for failing at least one of the two dominant DCE tasks. No participant completed the DCE tasks within 150 s (minimum time: 174 s). Therefore, the remaining 1,001 participants were included in the analysis. For the C-TTO, 205 adult participants responded. Two participants were excluded because all observed utility values for the 10 health states were 1 as this indicated non-participation in the C-TTO tasks; thus, the remaining 203 participants were included in the analysis. Table 1 shows the characteristics of the study sample. The age, sex, and geographical distribution of the participants matched the population distribution in the 2021 Population Census of Hong Kong [13].

**Table 1:** Characteristics of the study sample

	DCE sample		C-TTO sample		Hong Kong population distribution*
	*N*	%	*N*	%	%
**Age**					
18–39 years	384	38.4	83	40.9	31.1
40–59 years	387	38.7	64	31.5	36.9
60 years or above	230	23.0	56	27.6	32.1
**Sex**					
Female	568	56.7	112	55.2	55.3
Male	433	43.3	91	44.8	44.7
**Education** ^**+**^					
Primary or below	54	5.4	2	1.0	18.4
Secondary	289	28.9	54	26.6	47.0
Post-secondary	658	65.8	146	72.0	34.6
(missing)	0	0.0	1	0.5	
**Marital status**					
Never married	354	35.4	81	39.9	
Married/ Cohabited	569	56.8	103	50.7	
Widowed/ Divorced/ Separated	78	7.8	18	8.9	
(missing)	0	0.0	1	0.5	
**Any children**					
N	508	50.8	111	54.7	
Y	493	49.3	91	44.8	
(missing)	0	0.0	1	0.5	
**Any children below 18 years**					
N	812	81.1	168	82.8	
Y	189	18.9	34	16.7	
(missing)	0	0.0	1	0.5	
**Work status**					
Full-time	635	63.4	113	55.7	
Part-time	86	8.6	15	7.4	
Unemployed	13	1.3	4	2.0	
Student	91	9.1	22	10.8	
Housewife/ Retired	176	17.6	48	23.6	
(missing)	0	0.0	1	0.5	
**Household income per month (HK$)**					
< 10,000	69	6.9	13	6.4	
10,000–19,999	103	10.3	14	6.9	
20,000–29,999	162	16.2	25	12.3	
30,000–39,999	151	15.1	40	19.7	
40,000–49,999	125	12.5	32	15.8	
50,000–59,999	114	11.4	20	9.9	
> 60,000	277	27.7	58	28.6	
(missing)	0	0.0	1	0.5	
**Any chronic disease diagnosis**					
N	688	68.7	143	70.4	
Y	313	31.3	59	29.1	
(missing)	0	0.0	1	0.5	
**Living district**					
Hong Kong Island	114	11.4	19	9.4	16.3
Kowloon	314	31.4	55	27.1	30.1
New Territories	573	57.2	129	63.5	53.6
**Total**	1001	100.0	203	100.0	

∗ Census and Statistics Department. 2021 Population by Sex, Age, Year and District Council District, HKSAR, 2022

† Data for Hong Kong Census refers to population ≥ 15 years and for study sample ≥ 18 years

### C-TTO outcomes

**Fig. 1:**
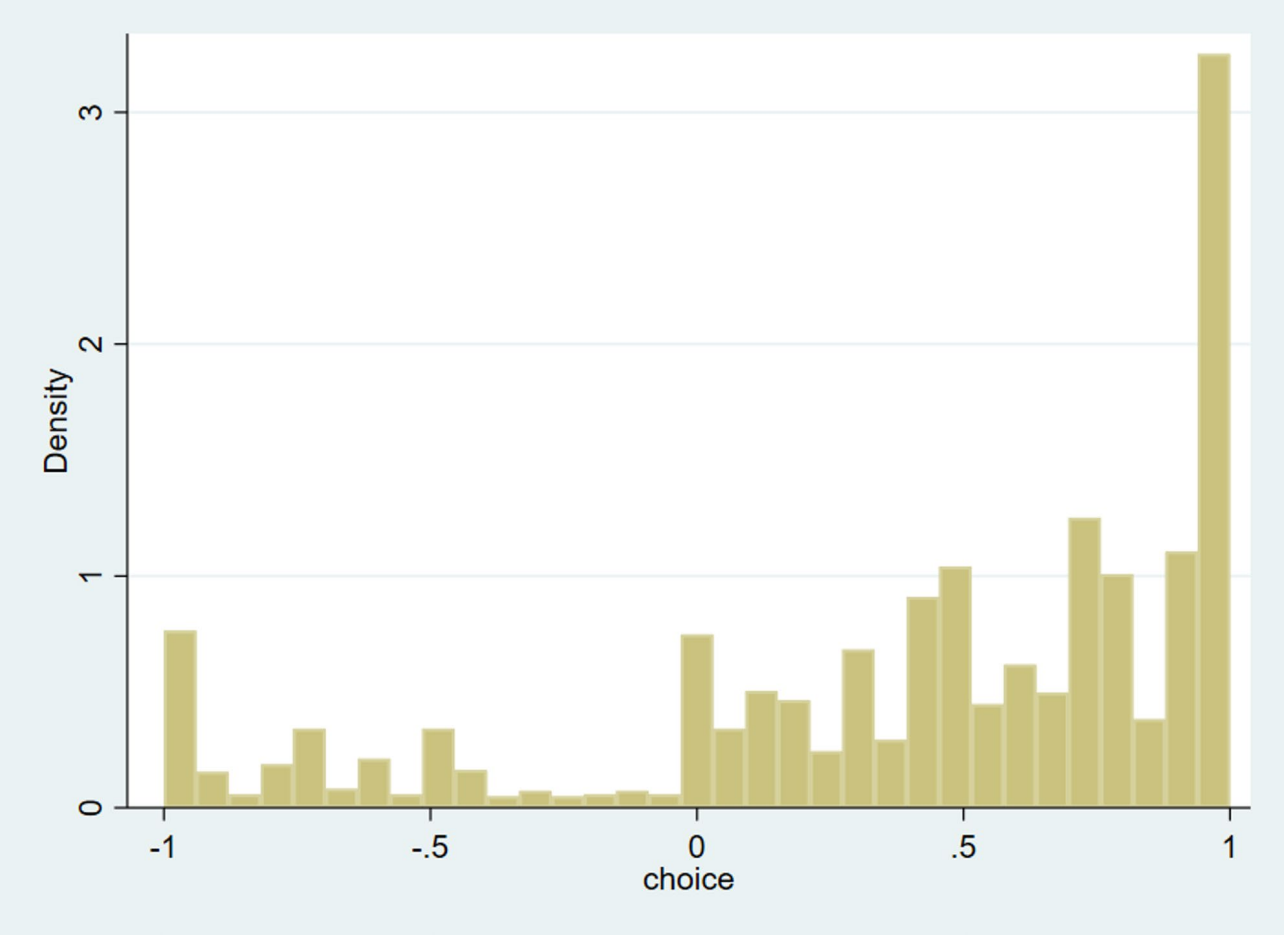
Distribution of the observed C-TTO values

The 203 participants with valid responses took a median of 29.5 min (IQR: 26.0–34.4 min) to complete the interview. Figure 1 shows the distribution of the observed C-TTO values. Among the responses to the 10 health states, 16.5% of the observed values (*n* = 334) were negative, and 4.2% (*n* = 86) of the values were − 1. Regarding the mean observed values by health states (Table 2), the highest values (0.919) were observed in state 11112, while the lowest values (-0.232) were observed in state 33333. Among the 10 states, state 33333 was the only one with an observed C-TTO value lower than 0. When comparing 11121 vs. 11112 and 22232 vs. 22223, pain/discomfort was considered more important than feeling worried, sad or unhappy. In the comparison of states 33233 vs. 33323, level 3 of pain/discomfort was considered more important than level 3 of doing usual activities. For the adjusted C-TTO utility, the value of state 33333 was − 0.258, and both states 33,333 and 33233 had negative values.

**Table 2:** Description of observed C-TTO utility

State	Observed utility		Adjusted utility by Tobit model	
	Mean	Std. Err	Mean	Std. Err
11112	0.919	0.009	0.919	0.009
11121	0.902	0.008	0.902	0.008
21111	0.907	0.012	0.907	0.007
22223	0.492	0.032	0.488	0.027
22232	0.382	0.034	0.378	0.031
31133	0.235	0.037	0.229	0.034
32223	0.402	0.032	0.398	0.027
33233	0.009	0.036	-0.003	0.033
33323	0.058	0.037	0.048	0.033
33333	-0.232	0.037	-0.258	0.035

### DCE outcomes and the value set

The outcomes of the DCE modelling are presented in Table 3. The BIC of the MIXL models was lower than that of the MNL models, likely because the MIXL models account for preference heterogeneity. The MIXL models with an ASC to indicate the left-right position of the alternatives presented a lower BIC and a higher log-likelihood than the MIXL model without such an ASC. Therefore, the MIXL model with the ASC was selected for generating the value set. In this model, all the EQ-5D-Y-3 L dimension levels were statistically significant at the 0.05 level.

**Table 3:** DCE modelling coefficients and anchored value set

choice	MNL model		MIXL model		Value set (regular dummy coding)*
	Coeff (SE)	Coeff (SE)	Coeff (SE)	Coeff (SE) (selected)	
*Mean*					
ASC		0.03 (0.02)		0.04 (0.03)	
mo2	**-0.47 (0.06)**	**-0.47 (0.06)**	**-0.60 (0.07)**	**-0.61 (0.08)**	0.0641
mo3	**-1.60 (0.09)**	**-1.60 (0.09)**	**-1.82 (0.12)**	**-1.79 (0.12)**	0.1891
sc2	**-0.18 (0.05)**	**-0.17 (0.05)**	**-0.21 (0.06)**	**-0.18 (0.06)**	0.0190
sc3	**-1.09 (0.07)**	**-1.09 (0.07)**	**-1.32 (0.09)**	**-1.31 (0.08)**	0.1380
ua2	**-0.67 (0.04)**	**-0.67 (0.04)**	**-0.70 (0.05)**	**-0.69 (0.05)**	0.0729
ua3	**-1.97 (0.06)**	**-1.95 (0.06)**	**-2.32 (0.08)**	**-2.28 (0.08)**	0.2405
pd2	**-0.81 (0.04)**	**-0.81 (0.04)**	**-0.92 (0.05)**	**-0.91 (0.05)**	0.0955
pd3	**-2.47 (0.07)**	**-2.47 (0.07)**	**-3.47 (0.13)**	**-3.42 (0.12)**	0.3606
ad2	**-0.60 (0.04)**	**-0.59 (0.04)**	**-0.71 (0.05)**	**-0.70 (0.05)**	0.0736
ad3	**-1.93 (0.06)**	**-1.93 (0.06)**	**-2.54 (0.10)**	**-2.58 (0.10)**	0.2719
*SD*					
ASC				0.06 (0.09)	
mo2			-0.05 (0.12)	-0.04 (0.11)	
mo3			**1.38 (0.09)**	**-1.45 (0.09)**	
sc2			**0.15 (0.13)**	**0.26 (0.10)**	
sc3			**-0.55 (0.10)**	**0.33 (0.12)**	
ua2			**0.08 (0.18)**	-0.10 (0.13)	
ua3			**0.45 (0.11)**	**-0.57 (0.11)**	
pd2			0.09 (0.10)	0.07 (0.08)	
pd3			**1.63 (0.13)**	**1.54 (0.11)**	
ad2			-0.10 (0.09)	-0.03 (0.08)	
ad3			**1.37 (0.09)**	**1.50 (0.09)**	
N (choice)	15,012	15,012	15,012	15,012	
N (participant)	1001	1001	1001	1001	
LL	-6452.591	-6451.647	-5939.051	-5926.77	
BIC	13008.28	13016.7	12084.3	12080.35	

*The rescaled anchoring approach with the lowest MAE and RSME would be chosen as the value set

Different value sets were generated by rescaling the latent DCE values using the C-TTO results through three approaches. Among these, the linear mapping method achieved the lowest MAE (0.0269) and RSME (0.0323) compared with the worst state anchoring (MAE: 0.0465; RSME: 0.0598) and the hybrid model (MAE: 0.0274; RSME: 0.0350), which indicates the best fit to the criteria. Therefore, the rescaled linear mapping approach was chosen as the final value set. The value set is shown in Table 3. The utility formula can be specified as:

$$\eqalign{ & U\left( {HS} \right) = 1 - 0.0641*MO2 - 0.1891*MO3 \cr & - 0.0190*SC2 - 0.1380*SC3 - 0.0729*UA2 \cr & - 0.2405*UA3 - 0.0955*PD2 - 0.3606*PD3 \cr & - 0.0736*AD2 - 0.2719*AD3 \cr} $$

For health states other than the full health (11111), the values ranged from 0.981 (12111) to -0.200 (33333). The most important dimension was having pain/discomfort (PD), followed by feeling worried/sad/unhappy (AD), mobility (MO), doing usual activities (UA), and looking after oneself (SC). There are 6 health states with a negative utility (2.5%), including state 31333, 13333, 32333, 33233, 23333, and 33333.

## Discussion

By adopting the published EQ-5D-Y-3 L valuation protocol, this study obtained the value set for this instrument for the Hong Kong population using a DCE to elicit adolescents’ preferences for the five dimensions and levels, and a C-TTO to anchor the DCE modelling outcomes on the EQ-5D-Y-3 L utility scale. To our knowledge, this is the first study to generate the EQ-5D-Y-3 L value set in Hong Kong, China. This study also applied and compared three different anchoring approaches in generating the value set, and the result suggested that the linear mapping approach resulted in higher predictive accuracy for the C-TTO health states compared with worst state anchoring and hybrid modelling.

The EQ-5D-Y-3 L dimensions shared the same relative importance in the MIXL modelling outcome and the value set, as the linear mapping approach rescaled all the preference weightings using the same scaling parameter. It was observed that the most important dimension was having pain/discomfort (PD), followed by feeling worried, sad, or unhappy (AD), doing usual activities (UA), mobility (MO), and the least important dimension was looking after oneself (SC). This relative importance ranking was the same as those identified in the Netherlands, Belgium, Japan, and Slovenia [19–22], and it was similar to the rankings in Germany [23] (PD, AD, UA, SC, MO; the order of SC and MO switched compared to Hong Kong), Hungary [24] (PD, AD, MO, UA, SC; MO and UA switched), Spain [18] (PD, AD, MO, UA, SC; MO and UA switched). Compared with the value set in mainland China [25] (PD, MO, UA, SC, AD), the ranking of the dimensions was the same, except that AD was ranked as the least important dimension in mainland China, while it was the second most important dimension in Hong Kong. Similar findings were also observed in comparison with Indonesia [26], where the ranking was PD, MO, UA, AD, and SC, indicating the AD was of less importance compared to the observations in Hong Kong. This disparity may reflect very different views of mental health and mood issues, particularly those affecting children and adolescents, among the general public in different countries and regions. The ratio of the highest level 3 weight to the lowest level 3 weight in Hong Kong was 2.61 (PD/SC), which is lower than the ratio in Indonesia [26] (3.85), Germany [23] (3.42) and Belgium [20] (2.80), similar to the Netherlands [19] (2.61), while higher than in many countries, including Hungary [24] (2.54), Spain [18] (2.37), Slovenia [22] (2.10), mainland China [25] (1.82), and Japan [21] (1.27). This suggests that the level of preference concentration on a single dimension was at a medium level among the published value sets.

In terms of the EQ-5D-Y-3 L worst state values, the utility for state 33,333 was − 0.200 in Hong Kong, which is higher than that in several European countries (Belgium: -0.475, Germany: -0.283, Hungary: -0.485, the Netherlands: -0.218, Slovenia: -0.691, and Spain: -0.539) [18–20, 22–24]. However, compared with Hong Kong, studies in other Asian populations reported positive utility values for the worst state, including mainland China (0.097), Indonesia (0.289), and Japan (0.288) [21, 25, 26]. Similarly, there were 6 health states (2.5%) with negative utility values in Hong Kong value set, which is lower than those in the Netherlands (3.3%), Germany (6.6%), Spain (16.1%), and Slovenia (20.6%), but higher than those in Asian countries where there were no negative utility values [18–26]. The differences in the worst state value and the percentage of health states deemed worse than death between Asian and European populations imply a potential cultural difference, indicating that the general public in Asia may view severe health states as more tolerable. The population in Hong Kong, influenced by both Eastern and Western cultures due to historical reasons, appears to lie in the middle among these countries and regions.

According to the findings, the relative importance of the health dimensions differed between child and adult populations. In the adult value set, MO was considered the most important dimension, followed by PD, SC, AD and UA [27]. The utility value of the worst state 55,555 is -0.864, which was much lower than the worst state 33,333 (-0.200) in the children. These differences suggested that people in Hong Kong perceive the health status differently for adults and children. MO and SC were considered relatively more important for adults, possibly because respondents believe that limitations in mobility and self-care ability among children can be compensated by caregivers. Differences may also reflect variations in the descriptive systems of the two instruments, such as “feeling worried, sad, and unhappy” in children versus “anxiety/depression” in adults as well as differences in modelling and anchoring methods, where the EQ-5D-5 L in Hong Kong was derived based on a hybrid model for DCE and C-TTO responses [27]. The disparity in values between the two instruments may highlight the need for a separate value set for children and adolescents. Nevertheless, the fact that EQ-5D-Y-3 L values were obtained based on adults’ preference for health states of another imaginative individual rather than themselves could affect the values. Currently, studies have been conducted to compare the preferences elicited from adolescents with those from adults and explore whether adolescents can indicate their preferences in the valuation exercise for themselves [15, 28]. However, there is no guideline available for the use of adolescent-based preference data in EQ-5D-Y-3 L valuation, and further studies are needed.

We applied three different anchoring methods according to the work by Mott et al. [17] and selected linear mapping for generating the value set. By using this approach, the value set is always aligned with the relative importance elicited from DCE, as it is derived by multiplying a consistent rescaling parameter for the coefficients of all dimensions and levels. This approach was adopted by the EQ-5D-Y-3 L valuation exercise in several countries, including Germany, Hungary, Japan, and the Netherlands. Compared with linear mapping, while the worst state approach is relatively straightforward and also maintains the DCE-based relative importance between the dimensions in the EQ-5D-Y utility values, it only uses the observed utility of one health state (i.e., the worst state 33333) for anchoring. As for hybrid modelling, the value set may be affected by disparities in sample size and preferences between DCE participants and C-TTO participants. Therefore, the linear mapping approach was selected in this study.

One of the key strengths of this study was that the DCE was conducted face-to-face with an interviewer available, rather than an online self-administered survey. This data collection method improves the quality of DCE data by enabling respondents to better understand their tasks and the choice sets, and by reducing serial non-participation in the DCE. Consequently, the vast majority of respondents (97.2%) provided valid responses to the two quality-checking choice tasks and no one completed the DCE tasks within 150 s. Regarding the limitation of the study, there was a larger proportion of respondents with post-secondary education attainment compared to the population distribution in Hong Kong, which is frequently observed in the valuation of different quality-of-life instruments in various countries [19. 22, 26]. A potential reason could be that individuals with higher education levels might be more visible to the research teams or the survey companies conducting the sample recruitment.

## Conclusion

This study presents an EQ-5D-Y-3 L value set for the Hong Kong children and adolescents population, following the international valuation protocol of this instrument. The most important health dimension was having pain/discomfort, followed by feeling worried, sad, or unhappy, doing usual activities, mobility, and looking after oneself. This value set enables future use of the EQ-5D-Y-3 L for measuring health-related quality of life and conducting economic evaluation for healthcare policies, services, and interventions used for children and adolescents in Hong Kong.

## Data Availability

All data generated or analysed during this study are available from the authors upon reasonable request.
